# Reduced Renal Uptake of Various Radiopharmaceuticals with Sodium Paraaminohippurate Coadministration in a Rat Model

**DOI:** 10.2967/jnumed.124.268411

**Published:** 2025-05

**Authors:** Marian Meckel, Stefanie Ehrenberg, Theresa Schmidt, Philipp Ritt, Margret I. Moré, Ralf Bergmann, Domokos Mathe, Konstantin Zhernosekov

**Affiliations:** 1 ITM Medical Isotopes GmbH, Garching, Germany;; 2 ITM Oncologics GmbH, Garching, Germany;; 3 Chair for Clinical Nuclear Medicine, Fiedrich-Alexander-Universität Erlangen-Nürnberg, Erlangen, Germany;; 4 Department of Biophysics and Radiation Biology, Semmelweis University Budapest, Budapest, Hungary; and; 5 CROmed Translational Research Centers Ltd., Budapest, Hungary

**Keywords:** Wistar rats, nephroprotective agent, paraaminohippurate, PAH, [^177^Lu]Lu-DOTATOC, radiopharmaceutical

## Abstract

The coinfusion of amino acids with targeted radiopharmaceutical therapy aims to reduce renal toxicity. Unfortunately, this requires a prolonged, large-volume infusion and often results in side effects such as nausea, vomiting, and hyperkalemia. Sodium paraaminohippurate is a nontoxic compound that has historically been used to measure renal plasma flow. It is excreted by the kidneys via glomerular filtration and tubular secretion using organic anion transporters. Paraaminohippurate has a favorable safety profile at plasma concentrations that saturate the maximum transport capacity of tubular cells. Therefore, paraaminohippurate may potentially reduce the renal accumulation of small-molecule radiopharmaceuticals. **Methods:** Preclinical studies, including ex vivo biodistribution, SPECT/CT, and PET analyses, were performed in Wistar rats to evaluate how coinjection of a paraaminohippurate solution affects the renal uptake of various radiopharmaceuticals compared with coinjection of a NaCl or arginine–lysine solution. **Results:** Paraaminohippurate was well tolerated, with no toxicity observed. Accumulated activity measured in the renal cortex was significantly lower for the small-peptide radiopharmaceuticals (0.9–2.5 kDa)—[^177^Lu]Lu-DOTATOC, [^177^Lu]Lu-DOTATATE, [^177^Lu]Lu-DOTA-JR11, [^177^Lu]Lu-DOTA-sargastrin, and [^177^Lu]Lu-DOTARGD—when paraaminohippurate was coinjected instead of NaCl. The renal uptake of [^177^Lu]Lu-DOTATOC, [^177^Lu]Lu-DOTATATE, and [^177^Lu]Lu-DOTA-JR11 was reduced by 46%, 83%, and 63%, respectively, at 1 h after injection with paraaminohippurate coinjection from the uptake after injection with NaCl. Kidney area-under-the-curve values were reduced by up to 60%, depending on the compound used. To a lesser extent, paraaminohippurate-mediated nephroprotection was observed with the prostate-specific membrane antigen (PSMA)–targeting molecules [^177^Lu]Lu-PSMA-I&T and [^68^Ga]Ga-PSMA-11. The renal uptake of the larger recombinant protein [^177^Lu]Lu-DOTA-Affiline-22 (18 kDa) and the folate derivative [^99m^Tc]Tc-etarfolatide was not affected. These in vivo imaging data were confirmed by ex vivo biodistribution studies. **Conclusion:** Coinjection of paraaminohippurate at a high concentration was found to significantly reduce the renal uptake of a select number of small-molecule radiopharmaceuticals. This indicates the importance of tubular secretion, as well as the potential role of anion transporters that may be saturated by a high paraaminohippurate plasma concentration. Therefore, paraaminohippurate comedication could serve as a fast, safe, and convenient alternative to amino acid infusion as a nephroprotective agent during targeted radiopharmaceutical therapy.

## Visual Abstract

**Figure:**
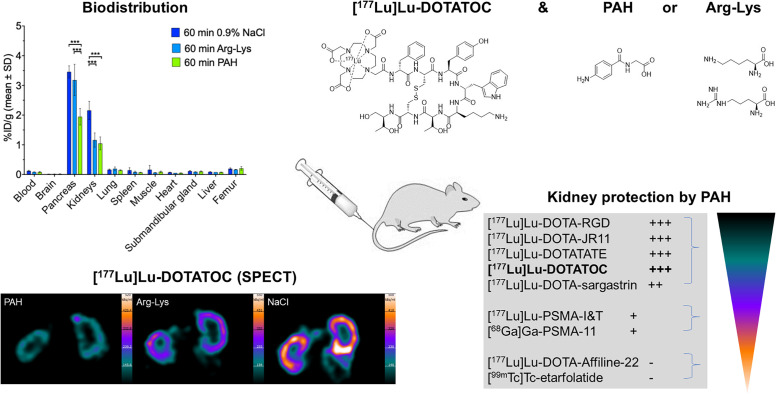

Different strategies have been investigated to reduce kidney uptake and the associated radiation burden during targeted radiopharmaceutical therapy (RPT) by coinfusion of agents that competitively inhibit the reabsorption of the radiolabeled compound, such as positively charged amino acids, Gelofusine (B. Braun Medical Industries), trypsinized albumin, or bovine serum albumin fragmented by cyanogen bromide (1,2). Other studies reported a more significant reduction in kidney uptake by using arginine–lysine (Arg-Lys) solution in combination with compounds such as amifostine and gelatin. To reduce the renal reabsorption of radiolabeled peptides more efficiently, a combination of 2 or more competitive renal transport inhibitors and other approaches may prove to be useful (3,4). Another approach uses radiolabeled prostate-specific membrane antigen (PSMA) inhibitors in combination with PSMA-binding molecules such as 2-(phosphonomethyl)pentanedioic acid or glutamate, which were found to improve the kidney-to-tumor ratio of radiolabeled PSMA-targeting molecules. The concept is based on the different kinetics of the involved molecules in kidneys and tumors (5).

For targeted RPT with radiolabeled somatostatin receptor agonist peptides, the coinfusion of amino acid solutions (AASs) as kidney radioprotectants is common practice but is frequently associated with adverse events, including nausea, vomiting, and hyperkalemia (6). In addition, the procedure requires 4–6 h, which could lead to a bottleneck in RPT wards.

Complete understanding of the excretion mechanisms of small-molecule–based radiopharmaceuticals, such as radiolabeled somatostatin analogs, remains elusive. Transport by megalin and cubilin has been identified as potentially relevant in the renal accumulation of radiolabeled peptides (7,8). In addition, it has previously been reported in rodent models that the inhibition of organic anion transporters (OATs) by probenecid significantly reduces the renal accumulation of [^111^In]In-DOTATOC. This indicates that OATs might also be involved in the renal clearance of somatostatin analogs (9). Furthermore, decreased accumulation of the amino acid 3-^125^I-iodomethyl-l-tyrosine in the renal cortex by blockage of the probenecid transporter system was reported (10).

Paraaminohippurate is a derivative of hippuric acid that has historically been used as a nontoxic diagnostic tool to measure the effective renal plasma flow, which serves as an indicator of renal function (11,12). The kidney filters more than 90% of paraaminohippurate from the plasma in a single pass-through, which is achieved by a combination of glomerular filtration and proximal tubular secretion. When the plasma concentration of paraaminohippurate is low, it is filtered by the glomeruli and almost entirely cleared from the renal bloodstream through active tubular secretion (13,14). However, increasing concentrations result in decreasing renal clearance, with increasingly nonlinear kinetics (15). At a concentration of 400–600 mg/L, saturation of the secretion mechanism of the tubular cells allows assessment of the functional capacity of the renal tubular secretory mechanism (16,17).

Paraaminohippurate serves as a substrate not only for OAT1 but also for several other renal transporters, such as OAT3 and OAT4, which transport various chemically unrelated compounds from the blood into the renal basolateral membrane, from which they are excreted into the urine (18,19). As an example, an increase in benzylpenicillin clearance was reported when administered with paraaminohippurate, suggesting a potential mechanism inhibiting both reabsorption and secretion processes in the kidney (20).

Recently, paraaminohippurate has undergone a head-to-head comparison with a standard Arg-Lys solution in 12 patients treated with [^177^Lu]Lu-DOTATOC. Application of paraaminohippurate was well tolerated and resulted in a significantly shorter treatment protocol while exhibiting kidney uptake of the radiolabeled compound comparable to that with the Arg-Lys solution (21). Thus, paraaminohippurate is a promising alternative to AAS as a kidney protectant during targeted RPT. In this study, we report the effect of paraaminohippurate coinfusion on the kidney uptake of several small-molecule–based radiopharmaceuticals, such as DOTATOC, DOTATATE, DOTA-JR11, DOTARGD, DOTA-sargastrin, PSMA-11, PSMA-I&T, and etarfolatide, as well as on the larger engineered protein DOTA-Affiline-22 (Navigo Proteins GmbH) in a rat animal model.

## MATERIALS AND METHODS

The supplemental materials give descriptions with additional details (supplemental materials are available at http://jnm.snmjournals.org).

### Chemistry and Radiolabeling

The paraaminohippurate solution was prepared by dissolving sodium paraaminohippurate (Sigma-Aldrich/Merck) in water for injection and adjusting the pH to 7 by using sodium hydroxide. 0.9% NaCl solution was purchased from Berlin-Chemie AG. DOTATOC, DOTA-sargastrin, DOTARGD, PSMA-I&T, and PSMA-11 were purchased from ABX. DOTATATE and DOTA-JR11 were obtained from Auspep and Pichem, respectively. Etarfolatide was purchased from BOCSCI Inc. DOTA-Affiline-22 was kindly donated by Navigo Proteins GmbH (22,23). Arginine and lysine were purchased from Sigma Aldrich/Merck KGaA and dissolved in water for injection.

Radiolabeling of the DOTA compounds was done at 85°C for 25 min, using ascorbic acid buffer and no-carrier-added [^177^Lu]LuCl_3_ in 0.04 M hydrochloric acid (ITM Medical Isotopes GmbH). The DOTA-Affiline-22 was labeled at 55°C for 1 h. Molar activity of the compounds was adapted based on the common human application range of 25–50 MBq/µg. Radiochemical purity was assessed by radio–high-pressure liquid chromatography (Agilent 1290 Infinity) and thin-layer chromatography (LabLogic Scan-RAM) with an acceptance criterion of more than 95%. ^68^Ga radiolabeling of PSMA-11 was done by eluting a ^68^Ge/^68^Ga generator (ITM Medical Isotopes GmbH) with 4 mL of 0.05 M hydrochloric acid into a 1-mL sodium acetate buffer containing 35 µg of PSMA-11. Radiochemical purity was assessed by thin-layer chromatography (LabLogic Scan-RAM) with an acceptance criterion of more than 95%. The ligand exchange method was used, with tartrate as a coligand for ^99m^Tc labeling. 100 µg of etarfolatide, 50 µL of tartrate solution (20 mg/50 µL in Millipore water), and 80 µL of stannous chloride dihydrate solution were added (1 mg/mL in 0.01 M hydrochloric acid solution) to approximately 750 MBq (20 mCi) of freshly eluted ^99m^Tc-pertechnetate. The reaction vial was incubated in a water bath for 30 min at 100°C and cooled to room temperature afterward (24).

### Animal Experiments

All animal experiments were conducted according to the European Union directive 2010/63/EU on the protection of animals used for scientific purposes, as well as in compliance with the Organisation for Economic Co-operation and Development’s Principles of Good Laboratory Practice. Approval for the work with laboratory animals was obtained from the appropriate animal care committee in compliance with the relevant national regulations. Animals were accommodated in standard housing conditions and were cage-acclimatized for at least 1 wk.

### Biodistribution

Experiments were conducted at the Helmholtz-Zentrum Dresden-Rossendorf, Institute of Radiopharmaceutical Cancer Research (approved by Landesdirektion Dresden, number DD24.1-5131/450/16, July 6, 2018). Groups of 5 male Wistar rats each (average body weight, 210 g) were randomized for coinjection of 0.9% NaCl, Arg-Lys solution (200 mg/mL), or paraaminohippurate solution (200 mg/mL). The animals received an intraperitoneal priming dose of 1.0 mL of 0.9% NaCl, 1.0 mL of Arg-Lys, or 1.0 mL of paraaminohippurate solution 10 min before radiotracer injection. The injection solutions for the biodistribution were prepared as mix of [^177^Lu]Lu-DOTATOC solution added to 1.5 mL of NaCl, Arg-Lys, or paraaminohippurate solution. The average injected activity ± SD of [^177^Lu]Lu-DOTATOC was 4.32 ± 0.51 MBq/kg in 4 animals, whereas 1 rat per group received 149 ± 15 MBq/kg.

As for paraaminohippurate, 500 mg paraaminohippurate were administered per rat; 200 mg thereof via intravenous injection; this by itself already results in an initial plasma concentration far exceeding the saturating 800 mg/L stated in (16). Because paraaminohippurate is rapidly excreted (16), an excessive dose was chosen and an intraperitoneal priming dose was administered as an additional depot.

Animals were killed 5 and 60 min after radiotracer injection; organs and tissues of interest were extracted, and their mass and activity were determined. Activities were counted in an automatic γ-counter, which was cross-calibrated with a dose calibrator by 3 standard samples. The activity of the tissue samples was decay-corrected and calibrated by comparing with the counts in the standard samples. Results were reported as percentage injected dose per gram of tissue (%ID/g).

### SPECT/CT

SPECT/CT experiments were conducted by CROmed Translational Research Centers Ltd. Healthy adult male Wistar rats (3 animals per group) received an intraperitoneal priming dose of 1 mL of 0.9% NaCl solution, 1 mL of paraaminohippurate (200 mg/mL), or 1 mL of Arg-Lys (200 mg/mL). Approximately 10 min later, 50–60 MBq of the ^177^Lu-radiolabeled radiopharmaceuticals were injected intravenously in combination with 0.5 mL of 0.9% NaCl, 0.5 mL of paraaminohippurate (200 mg/mL), or 0.5 mL of Arg-Lys (200 mg/mL). At 0.5, 1, 4, 8, and 24 h after injection of the ^177^Lu-radiolabeled compounds, SPECT/CT imaging was conducted under isoflurane anesthesia, using Mediso nano-SPECT/CT with a Silver upgrade system (Mediso Kft). VivoQuant version 1.22 software (VivoQuant) was used to determine the volume of interest and to quantify the activity in the kidneys. For obtaining the calibration factor, a solution of known volume and ^177^Lu-derived activity was imaged with the same parameter settings as for the rat SPECT/CT quantitative image acquisitions.

### PET

PET experiments were conducted by CROmed Translational Research Center Ltd. Healthy adult male Wistar rats (3 animals per group) received an intraperitoneal priming dose of 1 mL of 0.9% NaCl, 1 mL of paraaminohippurate (200 mg/mL), or 1 mL of Arg-Lys (200 mg/mL), followed by 15–30 MBq of [^68^Ga]Ga-PSMA-11 once intravenously in combination with 0.9% NaCl, paraaminohippurate, or Arg-Lys. PET examinations were conducted on an upgraded small-animal PET PRIMATE P4 scanner (Concorde Microsystems Inc.) at 5 min and 0.5, 1, 2, and 4 h after injection. As a reference, a solution of known volume and ^68^Ga-derived activity was placed in the field of view and imaged with the same parameter settings as for the rat PET quantitative image acquisitions.

### Statistical Analysis

Statistical analysis for the ex vivo organ distribution study was conducted using Microsoft Excel 2010 and GraphPad Prism version 6.05 (GraphPad Software). Statistical evaluation was performed using 2-way ANOVA. When significant overall effects were obtained by ANOVA, multiple comparisons were made with a Bonferroni multiple comparisons test. A *P* value of no more than 0.05 indicated a statistically significant difference.

## RESULTS

### Coadministration of Arg-Lys and Paraaminohippurate

**FIGURE 1.**
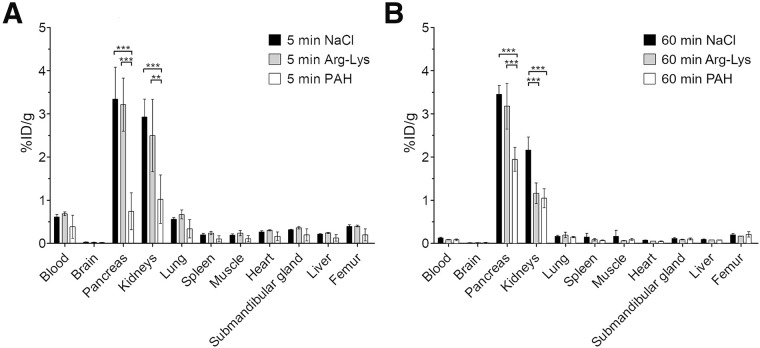
Biodistribution of [^177^Lu]Lu-DOTATOC at 5 min (A) and 60 min (B) after single intravenous injection delivered simultaneously with 0.9% NaCl, Arg-Lys, or sodium paraaminohippurate (PAH) solution in rats preconditioned with these compounds. Values are in %ID/g as mean ± SD (4 animals). **P < 0.01. ***P < 0.001. Other differences are not significant (*P* > 0.05). Exemplary values are in supplemental materials.

The coadministration of paraaminohippurate was well tolerated, and no immediate or delayed toxicity was observed in rats. Activity concentrations, measured in tissues during the ex vivo biodistribution study, are shown in Figure 1. Paraaminohippurate coinjection resulted in kidney uptake of [^177^Lu]Lu-DOTATOC of 1.02 ± 0.56 %ID/g at 5 min and 1.05 ± 0.22 %ID/g at 60 min, which was less than Arg-Lys coinjection (2.50 ± 0.84 %ID/g at 5 min and 1.16 ± 0.24 %ID/g at 60 min) and NaCl coinjection (2.93 ± 0.42 %ID/g at 5 min and 2.16 ± 0.31 %ID/g at 60 min). For other organs, no difference in uptake was observed, except for the pancreas, where uptake 5 min after injection reached 3.34 ± 0.74 %ID/g with coinjection of NaCl solution, which was more than the 3.22 ± 0.61 %ID/g reached with Arg-Lys coinjection and 0.74 ± 0.43 %ID/g reached with paraaminohippurate coinjection.

**FIGURE 2.**
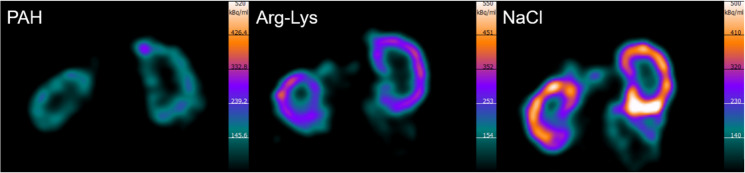
Exemplary SPECT images of kidney coronal sections 8 h after injection of [^177^Lu]Lu-DOTATOC in combination with paraaminohippurate (PAH), Arg-Lys, or 0.9% NaCl solution. Images indicate reduced radioactivity accumulation in renal cortex. Lighter color shows higher level of activity.

Exemplary SPECT images of kidney coronal sections 8 h after [^177^Lu]Lu-DOTATOC treatment in combination with paraaminohippurate, Arg-Lys, or NaCl are shown in Figure 2. The activity was predominantly retained in the renal cortex, and the concentration of [^177^Lu]Lu-DOTATOC when coinjected with paraaminohippurate was significantly lower than with NaCl and Arg-Lys coinjection (Table 1).

**TABLE 1.** %ID/g of Kidney Tissue Obtained from SPECT/CT Images of Male Wistar Rats After [^177^Lu]Lu-DOTATOC Coinjection and Changes Relative to 0.9% NaCl

Comedication	0.5 h	1 h	4 h	8 h	24 h
0.9% NaCl	13.3 (8.4)	12.2 (8.3)	2.7 (0.0)	2.7 (0.0)	2.4 (0.1)
200 mg/mL Arg-Lys	12.0 (1.6)	12.1 (3.6)	2.1 (0.5)	2.0 (0.4)	1.9 (0.4)
200 mg/mL PAH	6.6 (2.3)	6.6 (2.8)	1.4 (0.2)	1.2 (0.1)	1.2 (0.0)
Relative to 0.9% NaCl					
Arg-Lys	−10	−1	−22	−26	−21
PAH	−50	−46	−48	−56	−50

PAH = paraaminohippurate.

Values are presented as mean %ID/g with SD in parentheses or as percentages.

**FIGURE 3.**
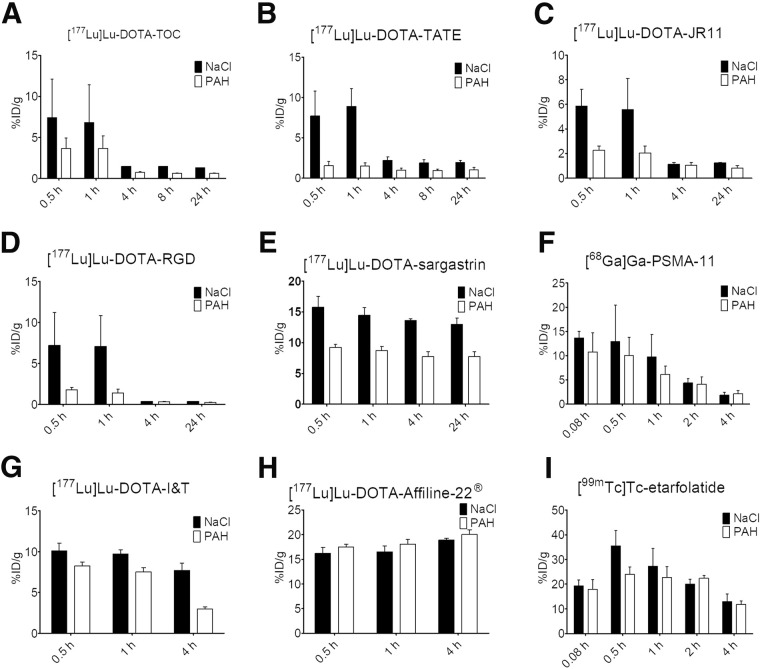
%ID/g of kidney tissue obtained from SPECT/CT or PET imaging of male Wistar rats after coinjection of various radiotracers with 0.9% NaCl solution (black) or paraaminohippurate (PAH) solution (200 mg/mL; white). Error bars show SD. Exemplary values are in supplemental materials.

Figure 3 shows %ID/g of kidney tissue obtained from SPECT/CT or PET imaging of the different radiotracers after coinjection of NaCl or paraaminohippurate solution. Independent of the coinjected compound, the radiolabeled small-peptide compounds [^177^Lu]Lu-DOTATOC, [^177^Lu]Lu-DOTATATE, [^177^Lu]Lu-DOTA-JR11, and [^177^Lu]Lu-DOTARGD (0.9–2.5 kDa), as well as the PSMA derivatives [^177^Lu]Lu-PSMA-I&T (1.5 kDa) and [^68^Ga]Ga-PSMA-11 (0.9 kDa), were absorbed by the kidneys, with peak activity levels at 0.5 h after injection, followed by a significant decrease up to 4 h after injection. Afterward, the renal uptake reached a steady state. A similar rapid uptake was observed for the peptide [^177^Lu]Lu-DOTA-sargastrin (2.5 kDa), with a small or almost negligible decrease over time. The renal excretion of [^177^Lu]Lu-PSMA-I&T followed a pattern similar to that of [^68^Ga]Ga-PSMA-11 but with a slower kinetic when coinjected with NaCl. For the preceding radiolabeled compounds, total renal uptake was always lower with paraaminohippurate coinjection than with NaCl coinjection; differences between paraaminohippurate and NaCl were most pronounced for small-peptide compounds.

The larger protein [^177^Lu]Lu-DOTA-Affiline-22 (18 kDa) showed maximum kidney uptake 24 h after injection regardless of the coinjected solution, although uptake was slightly higher when paraaminohippurate was used. The folic acid derivative [^99m^Tc]Tc-etarfolatide (0.9 kDa) showed a renal uptake profile different from that of the other compounds and independent of the coinjected solution. [^99m^Tc]Tc-etarfolatide achieved the highest renal uptake of all compounds studied: up to 36 %ID/g at 0.5 h after injection and a subsequent decrease to 13 %ID/g within 4 h after injection.

Table 2 provides an overview of the relative differences in the renal uptake of the compounds studied with paraaminohippurate coinjection compared with NaCl coinjection. The corresponding area-under-the-curve (AUC) values and relative changes obtained with paraaminohippurate coinjection are shown in Table 3.

**TABLE 2.** Relative Changes of Kidney Uptake of Various Radiolabeled Compounds with 200 mg/mL Paraaminohippurate Coinjection in Comparison to 0.9% NaCl

Compound	5 min	0.5 h	1 h	2 h	4 h	8 h	24 h
[^177^Lu]Lu-DOTATOC*	−65	ND	−51	ND	ND	ND	ND
[^177^Lu]Lu-DOTATOC	ND	−50	−46	ND	−48	−56	−50
[^177^Lu]Lu-DOTATATE	ND	−80	−83	ND	−56	−49	−46
[^177^Lu]Lu-DOTA-JR11	ND	−61	−63	ND	−10	ND	−35
[^177^Lu]Lu-DOTARGD	ND	−75	−80	ND	−14	ND	−29
[^177^Lu]Lu-DOTA-sargastrin	ND	−41	−40	ND	−43	ND	−40
[^177^Lu]Lu-PSMA-I&T	ND	−18	−22	ND	−61	ND	ND
[^177^Lu]Lu-DOTA-Affiline-22	ND	+8	+9	ND	+6	ND	+6
[^68^Ga]Ga-PSMA-11	−21	−23	−37	−5	+15	ND	ND
[^99m^Tc]Tc-etarfolatide	−7	−32	−16	+12	−8	ND	ND

* From biodistribution experiment.

ND = not determined.

Values are presented as percentages. Numbers with negative sign are reductions.

**TABLE 3.** AUC Values and Relative Changes of Listed Radiolabeled Compounds Under Paraaminohippurate (PAH) Coinjection in Comparison to NaCl

Compound	NaCl (%ID/g*h)	PAH (%ID/g*h)	Difference (%)
AUC_0–24 h_			
[^177^Lu]Lu-DOTATOC	87.0	43.0	−51
[^177^Lu]Lu-DOTATATE	113.7	45.4	−60
[^177^Lu]Lu-DOTA-JR11	72.5	46.4	−36
[^177^Lu]Lu-DOTARGD	47.0	18.7	−60
[^177^Lu]Lu-DOTA-sargastrin	577.9	338.5	−41
[^177^Lu]Lu-DOTA-Affiline-22	814.1	863.7	+6
AUC_0–1 h_			
[^177^Lu]Lu-PSMA-I&T	64.7	42.8	−34
[^68^Ga]Ga-PSMA-11	45.9	37.1	−19
[^99m^Tc]Tc-etarfolatide	152.3	140.7	−8

AUC_0–24 h_ = AUC within 24 h after injection; AUC_0–1 h_ = AUC within first hour after injection.

Paraaminohippurate coinjection resulted in a strong reduction in the renal uptake of small cyclic and linear peptides. In comparison, the reduction of PSMA ligand uptake was less pronounced. The renal uptake of the protein [^177^Lu]Lu-DOTA-Affiline-22 or the folic acid derivative [^99m^Tc]Tc-etarfolatide was not affected by paraaminohippurate.

## DISCUSSION

We investigated the effect of paraaminohippurate coinjection on the renal uptake of various radiolabeled small-molecule compounds. To achieve tubular saturation, paraaminohippurate was injected at the highest dose tolerated by the rats. The study cohort included ^177^Lu-labeled DOTA-conjugated cyclic and linear peptides, including DOTATOC, DOTATATE, DOTA-JR11, DOTA-sargastrin, and DOTARGD, as well as the peptidomimetic PSMA-affine compounds PSMA-I&T and PSMA-11. In addition, we investigated whether paraaminohippurate affects the renal uptake of the larger protein DOTA-Affiline-22 and the folic acid derivative [^99m^Tc]Tc-etarfolatide.

### DOTA Peptides

The greatest effect of a paraaminohippurate coinjection on renal uptake was observed with small cyclic and linear DOTA-conjugated peptides. For example, the renal uptake of [^177^Lu]Lu-DOTARGD was reduced by 80% 1 h after application, and [^177^Lu]Lu-DOTATATE showed a reduction of up to 83% when coinjected with paraaminohippurate from the uptake when coinjected with NaCl solution. Significant effects were also observed for [^177^Lu]Lu-DOTATOC (46% reduction), [^177^Lu]Lu-DOTA-JR11 (63% reduction), and [^177^Lu]Lu-DOTA-sargastrin (40% reduction; Table 2). These reductions were measurable at all time points, with the most pronounced effects within the first hour after injection. This is consistent with the rapid pharmacokinetics of paraaminohippurate.

For the previously mentioned compounds, renal AUC values for the 0- to 24-h period showed a strong decrease when paraaminohippurate was coinjected compared with NaCl solution. Similarly, the AUC within the first hour after injection was reduced by 36%–60%, depending on the small-peptide compound used, compared with 0.9% NaCl solution (Table 3). The results suggest that small linear and especially cyclic peptides are renally excreted not only via the megalin–cubilin pathway but also via OATs such as OAT1–OAT3. The influence of paraaminohippurate coinjection was particularly dominant in this cohort. Thus, paraaminohippurate may represent a safe and effective alternative to amino acid infusions, given that similar reductions in renal uptake were reported in patients with chronic kidney disease (21). Our data with 200 mg of Arg-Lys showed a reduction in renal DOTATOC uptake of 22% 4 h after injection (Table 1), in line with previously published data (16).

### PSMA Compounds

The renal uptake of PSMA-targeting compounds (PSMA-I&T and PSMA-11) was also reduced when combined with paraaminohippurate. However, the reductions observed were less pronounced than those seen with the other small DOTA peptides when combined with paraaminohippurate, because renal PSMA expression resulted in additional binding to the kidney cortex. Thus, unlike other radiolabeled small peptides such as DOTATATE or DOTATOC, coadministration of an AAS is not required in clinical practice during treatment with PSMA-targeting agents (25). However, reductions in the renal uptake of PSMA-targeting compounds when coadministered with paraaminohippurate suggest that OAT1–OAT3 appear to be involved in the elimination pathway of these compounds. The AUC values within the first hour after injection were reduced by 19% for [^68^Ga]Ga-PSMA-11 and by 34% for [^177^Lu]Lu-PSMA-I&T (Table 3).

### Larger Peptides and Proteins

Larger proteins are generally known to be retained in the kidney via the megalin and cubilin receptors within the proximal tubule. We showed that paraaminohippurate coinjected with 18 kDa of [^177^Lu]Lu-DOTA-Affiline-22 had no effect on renal uptake. This suggests that OAT1–OAT3 are not involved in the renal uptake of larger proteins. Therefore, [^177^Lu]Lu-DOTA-Affiline-22 could be considered a control for transport by megalin and cubilin receptors. Consistent with this, a significant reduction in renal uptake was observed when the smaller [^177^Lu]Lu-DOTA-sargastrin (17 amino acids long, 2.5 kDa) was used in combination with paraaminohippurate. Whether the total charge of the peptide, the amino acid sequence, or the size of the peptide plays a major role in these mechanisms remains unclear and was not investigated.

### Folic Acid Derivatives

Folate is a substrate for several transporters, including OAT1, and the folate analog methotrexate is a substrate for OAT3 (26,27). Therefore, changes in renal uptake might also be expected when using the folate receptor–targeting compound [^99m^Tc]Tc-etarfolatide in combination with paraaminohippurate. However, we did not observe strong changes in the renal uptake of [^99m^Tc]Tc-etarfolatide when used with paraaminohippurate. This could be because renal transport of folates is mediated by both proton-coupled folate transporters and the folate receptor.

The general limitations of this study are the low number of animals in each cohort, the limited number of time points, and the fixed amount of paraaminohippurate and treatment duration. In addition, we did not determine markers of kidney function or assess the impact of paraaminohippurate on tumor uptake in tumor-bearing rats.

## CONCLUSION

Preclinical studies conducted on Wistar rats demonstrated that the renal uptake of small-molecule radiopharmaceuticals, including [^177^Lu]Lu-DOTATOC, [^177^Lu]Lu-DOTA-JR11, and [^177^Lu]Lu-DOTATATE, was markedly diminished when these were coinfused with paraaminohippurate solution, as opposed to 0.9% NaCl solution. The AUC values for the kidneys were reduced, by up to 60%, depending on the compound used. Ex vivo biodistribution demonstrated no significant differences in [^177^Lu]Lu-DOTATOC uptake in organs besides the kidney and pancreas. This suggests that paraaminohippurate coinfusion may serve as a nephroprotective alternative to AAS coinfusion in a clinical setting (21). Paraaminohippurate may offer several advantages for patients receiving targeted RPT, including a lower infusion volume, a faster infusion time, and a reduction in adverse reactions. Therefore, paraaminohippurate may be a safer and more convenient option for patients than the currently used AAS. To demonstrate the potential for paraaminohippurate as comedication during targeted RPT, further research is required, including clinical dosimetry within carefully designed dose-finding studies.

## DISCLOSURE

The present work was funded by ITM Medical Isotopes GmbH and by the Hungarian National Research, Development and Innovation Office under the Research Grant Hungary scheme (contract number 151414 to Domokos Mathe). Within ITM Isotope Technologies Munich SE, Marian Meckel, Theresa Schmidt, and Konstantin Zhernosekov filed patent applications for the usage of paraaminohippurate with radiopharmaceuticals, WO2020225447A1, of which EP3965751B1 was granted, and for a combination of paraaminohippurate and radiolabeled complexes for treating cancer, WO2022096673A1, of which EP4065176B1 was granted. No other potential conflict of interest relevant to this article was reported.

## Notes

KEY POINTS **QUESTION:** How does a paraaminohippurate coinjection at a high concentration affect the kidney uptake of various radiopharmaceuticals in rats in comparison to coinjection of a 0.9% NaCl solution or an Arg-Lys solution? **PERTINENT FINDINGS:** In rats, the renal uptake of small-molecule radiopharmaceuticals such as [^177^Lu]Lu-DOTATOC, [^177^Lu]Lu-DOTA-JR11, and [^177^Lu]Lu-DOTATATE was significantly reduced when coinjected with paraaminohippurate solution from the uptake when coinjected with 0.9% NaCl solution. Depending on the compound used, AUC values for the kidneys were markedly decreased, by up to 60%. An ex vivo organ distribution experiment did not reveal significant differences in [^177^Lu]Lu-DOTATOC uptake in organs besides the kidneys and pancreas. **IMPLICATIONS FOR PATIENT CARE:** Paraaminohippurate, as a nephroprotectant, may offer several advantages for patients receiving targeted RPT, including a lower infusion volume, a quicker infusion time, and fewer adverse reactions.
